# Preliminary Study on Citrus Oils Antibacterial Activity Measured by Flow Cytometry: A Step-by-Step Development

**DOI:** 10.3390/antibiotics10101218

**Published:** 2021-10-07

**Authors:** Nayeli G. Nieto-Velázquez, Alejandra A. Gomez-Valdez, Marisela González-Ávila, Jaime Sánchez-Navarrete, Julia D. Toscano-Garibay, Nancy J. Ruiz-Pérez

**Affiliations:** 1 Dirección de Investigación y Enseñanza, Hospital Juárez de México, Ciudad de México 07760, Mexico; goretinieto@gmail.com; 2 Facultad de Estudios Superiores Cuatitlán, Universidad Nacional Autónoma de México, Cuautitlán Izcalli 54740, Mexico; alejandraanaid.gomezvaldez30@gmail.com; 3 Centro de Investigación y Asistencia en Tecnología y Diseño del Estado de Jalisco (CIATEJ), Guadalajara 44270, Mexico; mgavila@ciatej.mx; 4 Unidad de Banco de Sangre, Hospital Juárez de México, Ciudad de México 07760, Mexico; meyula@hotmail.com

**Keywords:** resistance, *E. coli*, flow cytometry, essential oils, citrus, viability

## Abstract

Essential oils of *Citrus sinensis* and *Citrus latifolia* have shown biological functions as antiseptics, anti-inflammatories, antioxidants, antifungal and antimutagenic, so the evaluation of their antibacterial capacity, by themselves or in combination with standard antibiotics, presents an alternative for infection treatment. Flow cytometry opens the door for the design of faster and more accurate measurement of antibacterial activity. We use a SYTO9/PI staining system on *E. coli* ATCC 25922 to determine antibacterial activity by counting live and dead cells through flow cytometry. We found that dual staining showed highly variable results due to wavelength overlapping and instead we used fluorochrome individual staining that highly correlated with viable counts. Chloramphenicol and cefotaxime treatments did not present a dose-response behavior, rendered diffuse readings and/or gave filament formation on fluorescence microscopy. Amikacin was a better comparison standard because it presented a dose-response behavior. Essential oils had low antibacterial activity as compared to amikacin, with a maximum of 10% and 20% for *C. latifolia* and *C. sinensis*, respectively. Combinations of essential oils with antibiotic resulted in an unforeseen strong inhibition of amikacin activity. Although a low antibacterial activity was found, a series of standardization steps are proposed for antibacterial activity measurement by flow cytometry.

## 1. Introduction

The World Health Organization has identified antibacterial resistance as one of the most dangerous threats to human health worldwide. The increasing number of resistant pathogens has compromised the efficacy of antibiotic stewardship and rates of associated morbimortality and economic burden have become seriously elevated in recent times [1]. This phenomenon has motivated the development of new antibacterial alternatives for both prophylactic and therapeutic purposes and particularly the research of those with a natural origin. Essential oils (EOs), by their chemical components, are presently considered as promising candidates to this end [2].

EOs are volatile oils produced by means of secondary metabolism of plants, mostly obtained by steam distillation or cold-pressing as opposed to plant extracts which are generated through the use of solvents like acetone, ethanol and/or hexane [3].

EOs are fragrant and rather transparent substances, while some are colorless when freshly distilled, yellowish when oxidized or colorful when obtained from flowers or other colored organs, ranging from pale yellow to green, and from blue to brownish red. EOs are highly soluble in lipids and organic compounds and are generally liquid at room temperature, although some could be solid or resinous [4,5]. They are a mixture of monoterpenes, sesquiterpenes, oxygenated derivatives, low weight alkanes, alcohols, aldehydes, ketones, esters and acids, associated with non-volatile paraffins and waxes [6]. EOs are present in all plant organs, including buds, flowers, leaves, seeds, stems, fruits, roots, wood and bark, although there is a general purveyance within secretory cells, cavities, channels and cells of the epidermis [7].

These oils are also known to have antiseptic activities as bactericidal, virucidal and fungicidal agents; medicinal properties as anti-inflammatory, sedative, neuroprotective, neuromodulator and spasmolytic bioactives, and even as antimutagenic and antioxidant [4,8].

It has also been reported that EOs have a wide range of inhibition against a large number of Gram-negative and Gram-positive bacteria. Their lipophilic nature makes them easily penetrative of bacterial cells, and therefore it has been observed that its mechanism of action is based on their ability to alter cell walls and the cytoplasmic membrane. This ability leads to lysis and loss of intracellular components, especially of ions [9], as well as generating a reduction in membrane potential, an interruption of proton pump and a depletion of ATP, among other consequences. It has also been established that the EO antibacterial effect may depend on a series of biochemical reactions typical of bacteria, which are associated with the proportion and type of oil constituents [10].

Also, EOs may produce an inhibitory effect by other mechanisms. It has been shown that its combination with other drugs generates an exceeding effect in comparison to their individual performance, generating greater antibacterial activity. This synergistic activity has been studied in order to reduce the antibiotic dose used for infection treatment. Some accepted theories to explain this phenomenon include the sequential inhibition of common bacterial biochemical pathways, inhibition of protective enzymes and its role as active cell wall agents to improve the diffusion of other antibacterial molecules [5]. 

On the other hand, *Citrus* genus is one of the most important crops in terms of world production, according to the Food and Agriculture Organization (FAO) of the United Nations [11]. It belongs to the *Rutaceae* family, which comprises around 140 genera and 1300 species. The best-known species are *C. aurantifolia* (lime), *C. hystrix* (kafir lime), *C. limonia* (mandarin lime), *C. limon* (lemon), *C. jambhiri* (hard lemon), *C. sinensis* (sweet orange), *C. aurantium* (bitter orange), *C. limetta* (sweet lime), *C. macroptera* (wild orange), *C. tachibana* (tachibana orange), *C. medica* (citron), *C. nobilis* (tangor), *C. paradise* (grapefruit), *C. reticulata* (mandarin) and *C. tangelo* (tangelo) [12]. This genus has played a very important role for centuries and has been used as additive in pharmaceutical, cosmetic and food industries. *Citrus* species contain abundant amounts of vitamin C and macronutrients including sugar, dietary fiber, potassium, folate, calcium, thiamine, niacin, vitamin B6, magnesium, riboflavin, and pantothenic acid. Additionally, there are a series of secondary metabolites, such as flavonoids, alkaloids, coumarins, limonoids, carotenoids, phenolic acids and essential oils that are mainly found within the multiple oil chambers on the peel of these fruits, conferring unique aromatic fragrances differentiative from each species and variety. Due to its composition, Citrus genus presents diverse important bioactivities for human health such as antioxidant, anti-inflammatory, cardiovascular protective effect, neuroprotective effect, etc. [13].

One of the most important species among this genus is *C. latifolia*, commonly known as Persian lemon, which has been used since ancient times for its antiseptic, carminative, diuretic and eupeptic effects [10]. Some of its components, such as β-caryophyllene, D-limonene and linalool, have shown anti-inflammatory effects; meanwhile α-pinene and β-pinene inhibit nitric oxide synthesis, suggesting an anti-oxidant role [14]. A recent study showed that β-pinene also exerts an antispasmodic effect in rat ileum and causes antinociception and that D-limonene have effects as antiulcerogenic, gastroprotective, chemoprotective, antiproliferative, insecticidal, antimicrobial, immunomodulatory and reduces symptoms of anxiety when use in aroma-therapy [10,14].

The juice of another member of this genus, *C. sinensis* or sweet orange, is the most consumed fruit juice worldwide for being an excellent source of vitamin C and for its properties as a natural antioxidant that strengthens the immune system [5]. It has been traditionally used to treat constipation, cramps, colic, diarrhea, cough, hypertension, anxiety, depression and stress [15]. Singh et al. [16] revealed the main components of two kinds of orange essential oil as D-limonene, follow by E-citral, Z-citral and carveol, which are recognized as natural antioxidants and food preservatives.

Ruiz et al. [17] demonstrated that the essential oils of *C. latifolia* and *C. sinesis* have good antifungal activity against different *Candida* species. *C. latifolia* EO was mainly composed of R-(+)-limonene (51.64%), β-thujene (14.85%) and γ-terpinene (12.8%), while *C. sinensis* EO was composed primordially R-(+)-limonene (96%) and α-myrcene (2.79%). Both oils were not genotoxic, that is, they do not produce mutations neither by reading frame displacement, base pair substitution, nor by ROS damage when evaluated through Ames test. Furthermore, *C. sinensis* EO did not show a cytotoxic effect on human buccal epithelium cells at doses used for the evaluation of its antifungal activity. However, the EO of *C. latifolia* was cytotoxic at its highest dose (21.8 μg). Additionally, Toscano et al. [18] demonstrated that these same oils have an antimutagenic effect against alkylating, polycyclic-aromatic and pro-oxidant mutagens.

Finally, in the early eighties the first studies were published on the application of fluorescent flow cytometry (FC) for the evaluation of antibacterial sensitivity. This technique has shown reproducible and statistically similar results as compared to those obtained by microdilution, but with performance times of <6 h in fast-growing bacteria and 16 h in slow-growing bacteria, such as *M. tuberculosis* [19]. Additionally, FC provides information about bacterial physiology through the evaluation of membrane permeability, metabolic activity and bacteria replication, allowing the detection of bacteria in a viable but non-culturable state (VBNC) which cannot be perceived in traditional methods [20]. FC determines bacterial viability through the reading of bacterial cells stained with specific fluorochromes that ultimately deliver information on such cell parameters. Different staining protocols have been developed, among them, SYTO9/Propidium Iodide (PI) dual staining has become the widest used protocol due to its fast-processing times and relative ease of use.

In here we aimed to evaluate the accuracy of SYTO9/PI dual staining in FC measurement of the possible antibacterial activity of *Citrus latifolia* and *Citrus sinensis* essential oils, simultaneously comparing the results to viable count method, with the purpose of establishing if FC could be a plausible test for the determination of bioactivity of these and other substances as alternatives or adjuvants of antibiotics.

## 2. Results

### 2.1. Standarization

#### 2.1.1. Flow Cytometer Sensibility and Determination of Detection Zones

**Figure 1:**
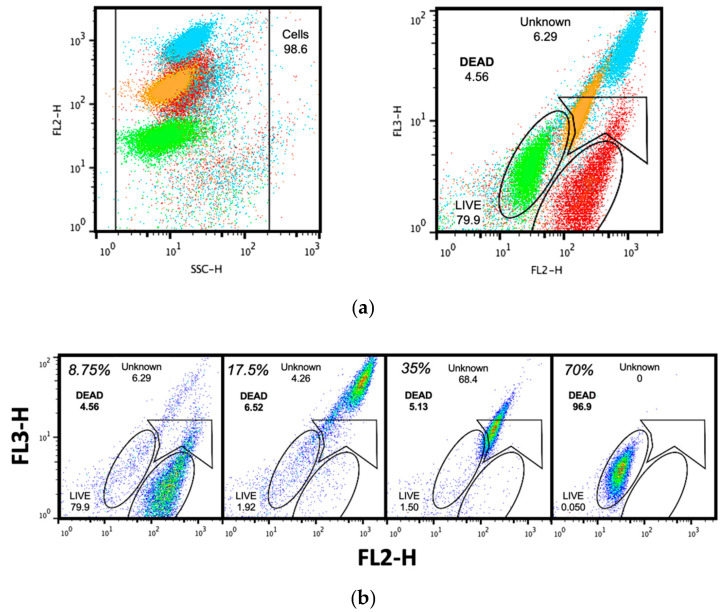
Dot plots of FC sensibility for dead cells detection. (**a**) Merged dot plots of red fluorescence (FL2-H) vs. side scatter (SSC-H) (left panel) and of green fluorescence (FL3-H) vs. red fluorescence (FL2-H) (right panel) for isopropanol dilutions at 8.75% (red), 17.5% (blue), 35% (orange) and 70% (green); (**b**) Dot plot of regions acquired from green fluorescence (FL3-H) vs. red fluorescence (FL2-H) for individual dilutions. Numbers represent the cell percentage with respect to the total acquired events.

In order to standardize the appropriate gating zone for bacterial populations and to determine the sensibility of the flow cytometer for measuring changes in dead cells quantities, a series of isopropanol dilutions were prepared, and selected gates are presented as dots in side scatter plot (Figure 1a, left panel) and fluorescence plot (Figure 1a, right panel). Data appeared in a distinctive area that was drawn accordingly to previous reports [21,22]. Briefly, two oval-shaped regions are drawn in parallel and with an upward inclination to delimit dead (upper oval) and live (lower oval) bacteria. A third region covering the upper part of the ovals was drawn to measure the number of cells in an unknown state. According to our results, gating parameters were accurately set for detection of bacteria cells. Noticeably, populations with low numbers of dead cells had a marked displacement from the expected selecting gates (Figure 1b) even reaching the region of live bacteria; hence, to avoid confusing readings, 70% isopropanol was preferred as dead cells control.

#### 2.1.2. Determination of Live and Dead CELL Populations

**Figure 2:**
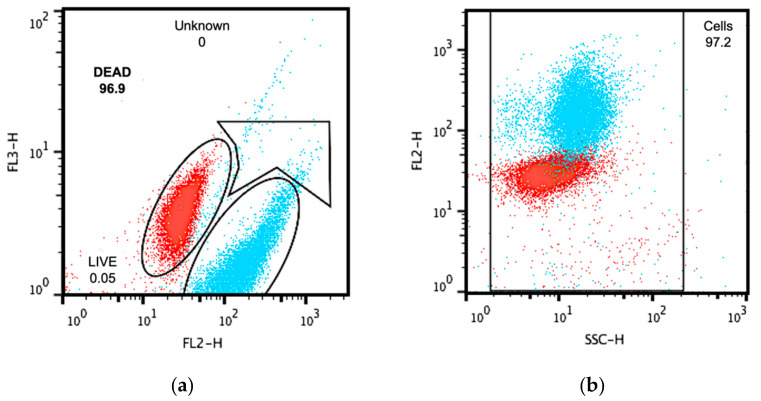
Dot plots of detection zones for live (blue) and dead (red) populations. (**a**) Dot plot of gates acquired from the red fluorescence (FL2-H) vs. side scatter or granularity (SSC-H); (**b**) Dot plot of gates acquired from the green fluorescence (FL3-H) vs. red fluorescence (FL2-H). Numbers represent the cell percentage with respect to the total acquired events.

To observe whether live and dead cells populations were located into the stablished detection zones within the FC dot plots, untreated and 70% isopropanol treated *E. coli* cultures were simultaneously stained with SYTO9/PI and read as described above. Figure 2 shows blue (live) and red (dead) dots that were inside the corresponding distinctive spaces in side scatter (Figure 2a) and fluorescence (Figure 2b) plots.

These treatments were denominated live (LCS) and dead (DCS) cell suspensions and constituted the live/dead controls that were prepared and simultaneously read in the experimental conditions used for EOs antibacterial determination.

#### 2.1.3. Standard Antibiotics

**Figure 3:**
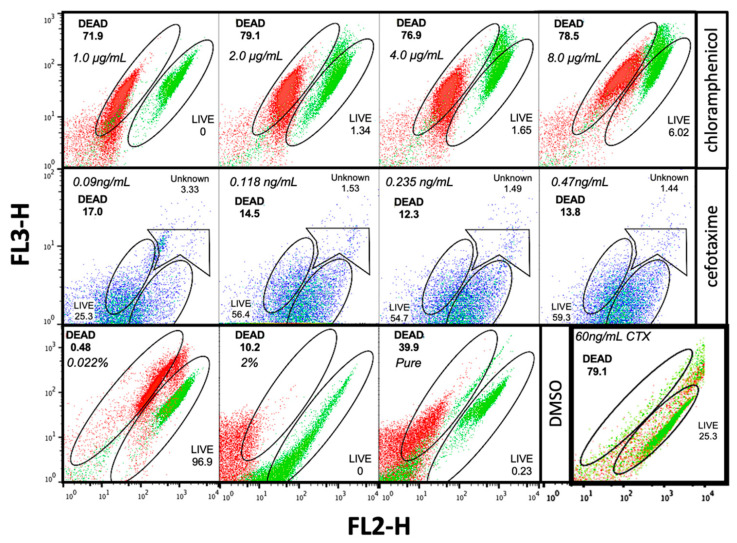
Standard antibiotics determination. Dot plots for the treatment of *E. coli* with chloramphenicol (upper panels), cefotaxime (middle panels) and DMSO (lower panels).

Once the cytometer was calibrated, *E. coli* cultures were subjected to antibiotic treatment in suspension (treated bacterial suspensions, TBS) as a preparative test for later comparison with experimental readings. Antibiotics and its concentrations recommended by the M100 file from the CLSI were used as starting point for cytometric examination under dual staining protocol. Figure 3 shows the resulting dot plots from chloramphenicol and cefotaxime treatments. Chloramphenicol does not render a number of dead cells that changes in accordance with the utilized concentrations (Figure 3, upper panels), hence it was not an eligible as reference antibiotic. Cefotaxime treatment, although highly recommended on the M100 file, had a rather diffuse behavior with dead/live populations undefinedly distributed along the whole plotting space (Figure 3, middle panels).

Higher concentrations of cefotaxime (60 ng/mL) seemed to redirect data of both populations into the selecting gates, nonetheless, dead and live cells regions still appeared mixed within the live cells zone (Figure 3, lower right panel).

Additionally, to elucidate if the dilutant present on the essential oils (DMSO) could interfere with the readings, two concentrations corresponding to the those used on experimental conditions and one treatment with pure DMSO were dot plotted using the same parameters. Results showed that populations treated with the dilutant were located within the expected areas and that even pure DMSO, which was not used in any experiment, had only moderate levels of toxicity on *E. coli* cultures (Figure 3, lower panels).

**Figure 4:**
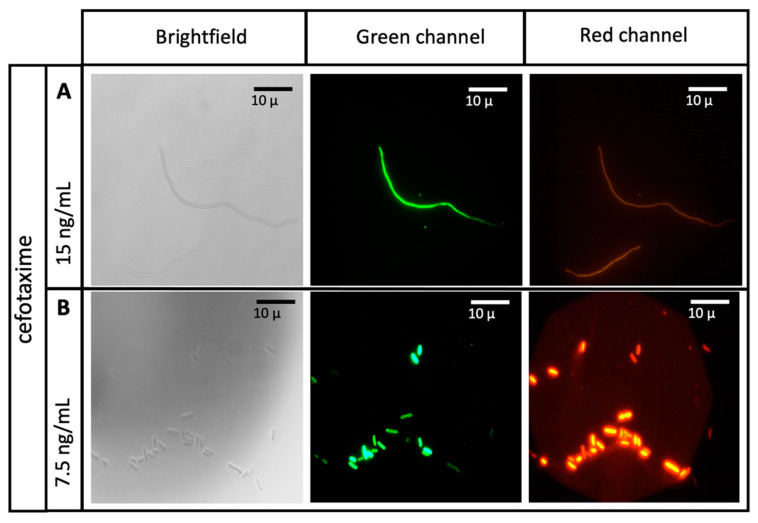
Epifluorescence microscopy (100×) of cefotaxime treated *E. coli* cells. (**A**), treatment at 15 ng/mL (**B**) treatment at 7.5 ng/mL.

Afterwards, to try to discern why live/dead populations readings were so disperse, cefotaxime treated cultures were stained with SYTO9/PI simultaneously and observed by epifluorescence microscopy. Figure 4 shows typical filament formation at a concentration of 15 ng/mL (Figure 4A) but not at 7.5 ng/mL (Figure 4B), images were acquired from the same field using three different channels. Filaments could not be properly measured using FC. It also can be seen that most bacteria were emitting both fluorescence colors, probably indicating an incomplete displacement of SYTO9 by PI. This phenomenon conducted to the production of a red-yellow emission causing the measurement of a single undefined population by FC that was determined as both live and dead cells. Based on these observations, we decided to test separate staining and reading both for EOs evaluation and for comparison antibiotic determination.

An antibiogram was then performed due to the previously observed resistance and erratic behavior of *E. coli* on cefotaxime treatment and in order to select a suitable antibiotic for reference. Amikacin was selected since this strain resulted sensitive on the agar diffusion test from among other antibiotics (Table 1).

**Figure 5:**
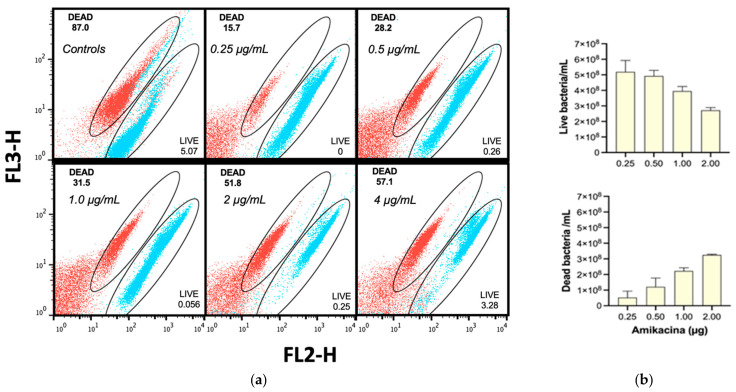
Dot plots of the standardization of live (blue) and dead (red) bacteria on amikacin treatment. (**a**) Representative dot plot of green (FL3-H) vs red (FL2-H) fluorescence of amikacin treatments; (**b**) Bar graphs of the calculated number of live and dead cells for amikacin doses. Bars are expressed as mean ± S.D.

Finally, several amikacin-treated *E. coli* cultures were stained independently with either SYTO9 or PI and then analyzed for cell populations. Figure 5 displays the resulting merged dot plots of an amikacin curve between 0.25 and 4 µg/mL, where it can be seen that live (blue) and dead (red) cells are adequately positioned within the selecting gates (Figure 5a) and that the quantization of dead and live cells (Figure 5b) had a direct correlation with the amikacin concentration used. Thus, amikacin was utilized as the comparison antibiotic for antibacterial evaluation of the essential oils.

### 2.2. Antibacterial Activity of Citrus Oils

**Figure 6:**
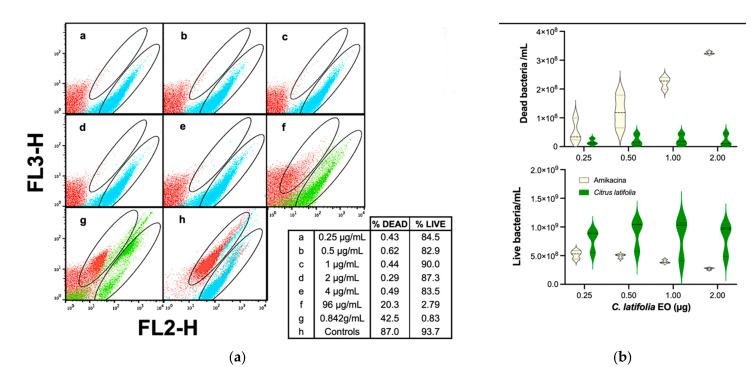
Evaluation of the antibacterial activity of *C. latifolia* EO. (**a**) Dot plot of green (FL3-H) vs. red (FL2-H) fluorescence readings for several concentrations of *C. latifolia* oil; (**b**) Violin graphs of the quantization of cell number for some concentrations used. Student’s *t*-test: *p* = 0.001 and *p* = 0.037 for live and dead bacteria vs. amikacin, respectively.

The essential oil of *C. latifolia* was evaluated using the FC parameters previously determined, carrying out separate staining and using the standardized comparison antibiotic (Figure 6), by means of a concentration curve between 0.25 μg/mL and 0.872 g/mL. From Figure 6a it can be seen that individual staining renders defined populations of live and dead cells, properly located on the expected gates, just as the pattern obtained on amikacin testing (Figure 5a). Although minimum inhibitory concentration was not reached, there was a dose-response behavior at the highest concentrations of the EO (Figure 6a,f–h). Subsequently, the number of live and dead cells was calculated and presented as violin graphs (Figure 6b) where a greater number of live cells can be observed as compared to amikacin treatment, this indicates that there was a poor antibacterial activity of *C. latifolia* EO at concentrations of 0.25 to 96 µg/mL.

**Figure 7:**
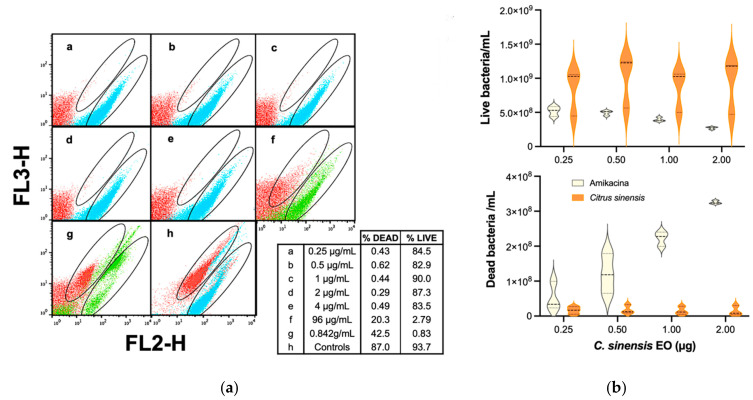
Evaluation of the antibacterial activity of *C. sinensis* EO. (**a**) Dot plot of green (FL3-H) vs. red (FL2-H) fluorescence gates for several concentrations of *C. sinensis* oil; (**b**) Violin graphs of the quantization of cell numbers in some of the used concentrations. Student’s *t*-test: *p* = 0.003 and *p* = 0.001 for live and dead bacteria, respectively.

Thereafter, essential oil of *C. sinensis* was evaluated under the same experimental conditions, but within a concentration curve from 0.25 μg/mL through 0.842 g/mL. Figure 7a shows well defined populations of live and dead cells within the selection gates. Minimum inhibitory concentration was not reached either, but a dose-response behavior similar to *C. latifolia* was observed at the highest concentrations (Figure 7a,f,g). Calculated numbers of live and dead cells are presented in Figure 7b, where the number of live cells on EO treatments exceed amikacin registers. These results indicated that there was a low antibacterial activity of *C. sinensis* EO at concentrations of 0.25 µg/mL to 4 µg/mL.

### 2.3. Combination of Citrus Oils with Amikacin

**Figure 8:**
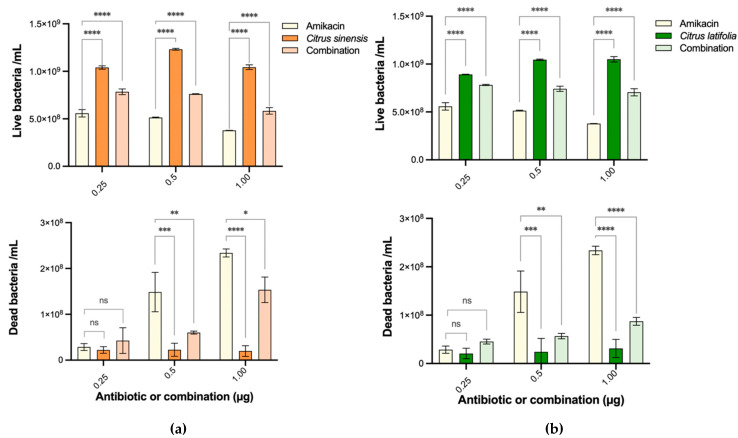
Evaluation of EOs combination with antibiotic. (**a**) Bar graph of the number of live and dead cells calculated from FC acquired gates for *C. sinensis* combined with amikacin. (**b**) Bar graph of the number of live and dead cells calculated from FC acquired gates for *C. latifolia* in combination with amikacin. Bars are expressed as mean ± S.D. 2-way ANOVA: ns = *p* > 0.05, * *p* ≤ 0.05; ** *p* ≤ 0.01; *** *p* ≤ 0.001; **** *p* ≤ 0.0001.

It has been reported that essential oils might not produce an inhibitory effect when used individually, but, when used in combination with standard antibiotics, EOs might present such effect or enhance the activity of the drug [23]. Combinations with several concentrations of amikacin were evaluated to examine a possible synergism, since citrus oils did not present an antibacterial activity by themselves at the evaluated concentrations (Figure 8). Graphs of the cell number count from FC showed that there was no synergic effect; on the contrary, EOs interfered with amikacin activity. This effect was produced in every concentration of amikacin used either with *C. sinensis* (Figure 8a) or *C. latifolia* (Figure 8b). 

### 2.4. Viable Counts

To corroborate the results obtained from FC, we randomly selected individual treatments and combination from the experimental set and performed viable count assays. The number of CFUs obtained for the selected sample confirmed a poor antibacterial activity for essential oils at any of the tested concentrations (Table 2). A higher number of colonies further confirmed the inhibition of amikacin activity in the presence of the oils.

## 3. Discussion

The use of natural origin compounds with antibacterial properties is an excellent alternative to avoid adverse effects of synthetic molecules and the bacterial resistance to them. A rapid and reliable diagnosis is a key strategy to reduce inappropriate antibiotic prescription, which is the main cause of antibacterial resistance [22,24]. Standard antibacterial susceptibility evaluation methods require long times to obtain results (24 h to 1 month). Due to this, different techniques have been developed to generate results comparable to those obtained by standard methods, but in less time, such as those obtained by the flow cytometry (6–16 h). We evaluated the antibacterial activity of citrus oils by this technique; however, we detected some limitations such as the observation of a region of intermediate states called “unknown population” [25] when counterstaining with SYTO9/PI, and a red-yellow fluorescence by microscopy, specifically in the treatments carried out with cefotaxime in *E. coli*.

Propidium iodide is a fluorophore with high affinity for DNA and RNA which only goes through intact permeable membranes and its combination with a fluorophore diffusing intact non-permeable membranes allows the counting of global cell number. Dual staining of SYTO9/PI is still widely used for the evaluation of antibacterial activity in different areas, nonetheless, its performance and effectivity are not yet clear and neither its effects on bacteria physiology. Several factors should be taken into account for a correct use of this dual staining system such as fluorochrome affinity, physiological state of cells to be stained and, incubation temperature and time. For instance, it is known that *E. coli* is more resistant to SYTO9 penetration than Gram positive bacteria due to cell membrane characteristics. Previous studies have shown that SYTO9 penetrates the cell in a continuous manner, meanwhile PI does it through continuous cytoadherence and that properties of the membrane could be altered by the penetration of one or both fluorochromes. On the other hand, false cell mortality signals could be displayed upon PI staining as a result of a high membrane potential, which might not correlate with culture observations due to a viable but non-culturable state (VBNC). Considering all these factors is of the uttermost importance for an accurate interpretation of FC results [26,27].

Besides, fluorochrome retention during the dynamic process of binding, release and competence for DNA binding sites [25] and the consequent increase in green fluorescence absorption by PI (fluorescence energy transfer or FRET) on suboptimal concentrations, could affect either fluorochrome or background signals. Hence, an adequate proportion with each other fluorochrome, and with the DNA content, could minimize such interference [26].

Filaments observed at low cefotaxime concentrations, could be due to the inhibition of PBP3 protein as an initial response in *E. coli* ATCC 25922. This protein is involved in peptidoglycan crosslinking during cell division, as described by Kjeldsen et al. in *E. coli* MG1655 [28]. However, at high cefotaxime concentrations, CTX-M-1 expression reached high levels, and the antibiotic was hydrolyzed, being unable to further bind to PBP3 [28].

MIC could not be obtained by flow cytometry because less than 80% of dead bacteria and a high rate of bacterial debris were detected at the highest evaluated concentration of amikacin. This could be due to the prolonged exposure time to the antibiotic which might have caused bacterial lysis; therefore, it is necessary to perform time-mortality curves to obtain optimal incubation times where antibiotic exerts its effect without disrupting bacterial integrity.

In regard to the treatment with essential oils, Frassinetti et al. [15] found an antibacterial activity from *C. sinensis* on *E. coli* by measuring cultures optical density, according to their results there was over 90% reduction in absorbance at 25 µg/mL, and raised the conclusion that time of exposure to EOs could be a determining factor to observe its antibacterial activity. Similarly, Dhiman et al. [29] used microdilution and disc diffusion method (DDM) to show that methanolic extract of *C. sinensis* peel had antibacterial activity on *E. coli* with a minimum inhibitory concentration of 0.78 µg/mL and a minimum bactericidal concentration of 6.25 µg/mL. On the other hand, Das et al. [30] showed that the methanolic extract of *C. latifolia* had antimicrobial inhibitory activity against E. coli in agar well diffusion method. Analogously, Everton et al. [31] found that the EO of *C. latifolia* had activity against *E. coli* and *S. aureus* on DDM.

In contrast, we observed that percentages ≥80% of dead bacteria were not obtained by flow cytometry, even at concentrations of 96 µg/mL, 8.720 mg/mL and 0.872 g/mL of *C. latifolia* EO and 96 µg/mL and 0.842 g/mL of *C. sinensis* EO.

Our results are more similar to those obtained by Adham et al. [32] who evaluated the antibacterial activity of peel ethanolic extracts and juice from four citrus species by DDM and found that *C. sinensis* had poor activity for Gram positive bacteria and null effect on Gram negative bacteria. Additionally, Tran et al. [33] results showed no activity of the *C. latifolia* EO on *E. coli* and other Gram negative bacteria such as *S. typhimurium* and *P. aeruginosa*, as opposed to the antibacterial effect observed on Gram positive bacteria like *Bacillus cereus*, *Listeria monocytogenes* and *Staphylococcus aureus*.

Although the results may seem divergent, it can be seen that, in general, citrus EOs have poor or no antibacterial effects against Gram negative bacteria, probably due to cell wall and membrane differences in contrast to Gram positive. Another difference between our results and the literature is the type of extraction, while we tested the essential oils from peel, other works were carried out using ethanolic extracts, methanolic extracts or juice. Differences have also been reported depending on the part of the plant from which these products are obtained, such as peel, seed, pulp, leaves, etc., which consequently yields multiple compositions that are unique on each research. On the other hand, it has been reported about the differences depending on the origin of the crops and the harvest time of the citrus fruits, among other intrinsic factors. Finally, it has also been reported that essential oils with high concentration of hydrocarbon terpenes may be more likely to have low antibacterial activity [5,34].

Despite these observations, most of the biological properties of *Citrus* genus have been attributed to limonene, one of its main components and a hydrocarbon terpene. For instance, in *Citrus sinensis Osbeck cv. Newhall* limonene is the main component (85.32%), and an antibacterial effect was shown at concentrations of 1.56 µL/mL, 0.78 µL/mL, 1.56 µL/mL and 3.13 µL/mL, against *S. cerevisiae*, *E. coli*, *B. subtilis* and *S. aureus*; respectively. However, when limonene was evaluated individually, MIC values were 15.68 µL/ mL (*S. cerevisiae* and *E. coli*) and 3.92 µL/mL (*B. subtilis* and *S. aureus*). In addition, authors observed that coexistent components at lower percentages within this EO, such as α-pinene, linalool, decanal, terpineol, citral and nonanal, presented higher antibacterial activity as compared to limonene [35]. Our results are congruent with this study since the EOs used are composed mainly by limonene and had poor antibacterial activity.

Factors such as functional groups of active components and their synergistic interactions will be decisive in the activity of EOs, since it has been shown that the effect of individual components differs from the one obtained in combination within the EOs, since each component exerts a different mechanism of action in the bacteria. This is the main reason for EOs to be proposed as new antibacterial compounds since it would be more difficult for bacteria to generate resistance to all of its components [35].

On the other hand, the decrease in the activity of amikacin in combination with EOs could be due to the alteration of its solubility by the presence of hydrophobic components (D-limonene, β-thujene, α-pinene, β-pinene, α-myrcene and γ-terpinene). The solubility of amikacin in water is 50 mg/mL and is much less soluble in DMSO (<1 mg/mL) present in the EOs dilutions [36], indicating that the solubility of amikacin decreases in the presence of organic solvents. 

Finally, although there are currently automated systems for the evaluation of antibacterial susceptibility that provide results in time ranges comparable to flow cytometry, this technique has the advantage of determining viability of culturable and nonculturable (VBNC) bacteria through measurement of parameters such as membrane integrity and metabolic activity. We found that evaluation of antibacterial susceptibility through dual staining presents certain limitations that were overcome by separation of stains.

## 4. Materials and Methods

### 4.1. Reagents and Strains

Reference strain *Escherichia coli* ATCC 25922 (Microbiologics^®^, Saint Cloud, MN, USA) was used as recommended by the CLSI. Mueller-Hinton broth and agar, Trypticasein-Soy agar, Nutrient agar and Blood agar were acquired from Becton Dickinson (BD, Franklin Lakes, NJ, USA). Soft agar was prepared with 0.6 g agar base (BD, Franklin Lakes, NJ, USA) and 0.6 g NaCl (Promega, Madison, WI, USA).

Saline solution (0.85% NaCl) (J.T. Baker, Phillipsburg, NJ, USA) was used for washing and maintaining cultures. 2% DMSO (J.T. Baker, Phillipsburg, NJ, USA) was used as dissolvent for essential oils. Antibiotics cloramphenicol, cefotaxime, and amikacin were purchased from AMSA (Coyoacan, CDMX, Mexico).

LIVE/DEAD^®^ BacLight™ Bacterial Viability and Counting kit (Cat. L34856, Thermo Fisher, Waltham, MA, USA) was used for sample staining.

McFarland turbidity standard was prepared by mixing 0.05 mL of 1.175% barium chloride dihydrate (BaCl_2_•2H_2_O), with 9.95 mL of 1% sulfuric acid (H_2_SO_4_). All standard preparations were verified in a Coleman spectrophotometer at 600 nm (abs. 0.063) previous to be used.

### 4.2. Essential Oils of Citrus sinensis and Citrus latifolia 

EOs of *C. sinensis* and *C. latifolia* were obtained by hydro-distillation of the peel, kindly donated by Frutech International Corporation Cargee Additives, Montemorelos Nuevo León, México, for Dr. Marisela González Ávila. Its composition was previously described by Ruiz et al. [17].

### 4.3. Calibrated Bacterial Solutions (CBS)

*E. coli* strain was seeded in trypticasein-soy agar for 24 h at 37 °C. Bacteria were then resuspended in SS and compared to 0.5 McFarland standard in a spectrophotometer (Junior II model 620, Coleman, Maywood, IL, USA).

### 4.4. Antibacterial Agents

Seven serial dilutions of cefotaxime were prepared by duplicate for flow cytometry measurements, and three dilutions for fluorescence microscopy, according to CLSI recommendations [37]. Five amikacin dilutions were prepared by duplicate to be used as CLSI recommended reference antibiotic. Essential oils of *C. latifolia* and *C. sinensis* were serially diluted in 2% DMSO to reach same concentration as amikacin, so bacterial susceptibility could be compared. Table 3 shows the whole set of preparations.

### 4.5. Combination Assays

For testing possible effects of EOs in combination with antibiotic, three dilutions of amikacin were mixed with 1 μg/mL of *C. sinensis* or *C. latifolia* essential oils as shown in Table 4.

### 4.6. Treated Bacterial Suspensions (TBS)

1 mL of 0.5 McFarland CBS was resuspended in 1 mL of Mueller-Hinton broth. Then, 250 μL of this suspension was added to either antibiotics, EOs alone or EOs + amikacin mixtures and incubated at 37 °C for 3 h under 70 rpm agitation. 

After incubation each sample or TBS was centrifuged at 470 g for 5 min and supernatant was discarded. Pellet was washed twice with saline solution (SS) and finally resuspended in 1 mL SS.

### 4.7. Flow Cytometry

Fluorochrome stock solutions were prepared by adding 3 μL of SYTO9 (5 mM) or 3 μL of PI (20 mM) to 97 μL of distilled water. 

For live (LCS) and dead (DCS) cell suspensions, 1 mL of CBS was centrifuged at 12,700× *g* for 5 min and resuspended either in 1 mL of SS for LCS or in 1 mL of 70% isopropanol for DCS. Staining was performed by mixing 178 µL of LCS or DCS with 10 µL of SYTO9 or PI stocks, respectively. Mixtures were incubated at room temperature for 15 min and read with the flow cytometer (FACScalibur, Beckton Dickinson, Franklin Lakes, NJ, USA).

Flow cytometer excitation laser was 488 nm in wavelength and fluorescence emissions were collected on red and green channels. LCS stained with SYTO9 and DCS stained with PI were used to locate the corresponding bacterial populations and to standardize most adequate cytometric parameters, avoiding emission spectra superposition. Frontal dispersion, lateral dispersion and fluorescence were acquired with logarithmic signal amplification. First, lateral and frontal dispersion amplifications were configured, followed by green fluorescence amplification to evidence live bacteria, and finally, red fluorescence amplification was tuned to show dead bacteria. Live and dead bacteria regions in the dot plots were defined using the parameters of SSC: 430, FL1: 659, FL2: 520 and FL3: 55. For dual staining, samples were prepared by mixing both dead and live suspensions (10 μL each) and incubating with SYTO9/PI (10 μL each) in SS for a final volume of 200 μL during 15 min at room temperature.

### 4.8. Fluorescence Microscopy

TBS was resuspended in 100 µL of SS with 10 µL SYTO9 and these mixtures were incubated for 15 min at room temperature before smearing them on slides (live bacteria). Similarly, isopropanol and cefotaxime treated samples were dual stained with SYTO9/PI to visualize fluorochrome acquisition and morphological changes using an Axio Imager.A2 microscope with an integrated camera (Axiocam ICc5, Carl Zeiss Microscopy GmbH, Jena, Germany).

### 4.9. Viable Count

10 µL of all TBS (amikacin, EOs, combinations and isopropanol) and untreated bacteria were resuspended in soft agar and immediately added to nutritive agar. After incubation for 24 h at 37 °C, the number of colony-forming units (CFUs) in each plate were counted on a colony counter (AccuLite Colony Counter Model 133-8002, Fisher Scientific International Inc., Hampton, NH, USA) and cell number were adjusted according to the dilution factor.

### 4.10. Statistical Analysis

Data were analyzed using GraphPad Prism and SPSS. Statistically significative differences between essential oils and amikacin were determined by student’s *t*-test. Values with *p* < 0.05 were considered significant. Comparisons of EOs in combination with amikacin were made through two-way ANOVA analysis, *p* < 0.05 were considered significant.

## 5. Conclusions

Flow cytometry is a useful tool in the evaluation of bacterial susceptibility because it shows reproducible results as compared with viable count technique, but with faster processing times. Even so, we consider that research with FC could also be focused on the development of new dual staining systems for bacterial viability since this field is still scarcely explored and existing stains have not been fully standardized, still having certain limitations. This was the case for SYTO9/PI dual staining, where the reliability of results was compromised by its inability to discriminate between viable and non-viable bacteria due to the displacement of populations (live and dead) towards an “unknown” region by the retention of both fluorochromes within cells.

Under the experimental conditions and staining settings found in this study, the essential oils of *C. latifolia* and *C. sinensis* presented poor antibacterial activity on *E. coli*, perhaps due to the short exposure time.

Interestingly, EOs presented an antagonistic effect against amikacin that may be due to a solubility change in the presence of organic components such as D-limonene, β-thujene, α-pinene, β-pinene, α-myrcene and γ-terpinene.

To our best knowledge, this is one of the fewest works that follows a series of systematic standardization steps to found adequate conditions for the flow cytometer acquiring of bacteria, fluorochrome concentration, determination of suitable comparison antibiotics and exposition times to antibiotics, to be used in the determination of antibacterial activity.

Even though this is a preliminary study where FC settings were standardized for a unique *E. coli* strain, the experimental development reported in here could be applied on future studies on Gram positive and/or Gram negative strains/species by contrasting the results against microdilution method. Additionally, it would be of interest to perform studies directed to prove the antagonism hypothesis of citrus EOs against amikacin.

## Figures and Tables

**Table 1:** Antibiogram for *E. coli *ATCC 25922.

Antibiotic	µg	Result
Amikacin	30	S
Ampicillin	50	R
Carbenicillin	2	R
Cephalothin	15	R
Cefotaxime	50	R
Ciprofloxacin	10	S
Chloramphenicol	300	R
Gentamicin	1	S
Netilmicin	10 U ^1^	S
Nitrofurantoin	30	R
Norfloxacin	25	R
Sulfamethoxazole/Trimethoprim	30	S
Ofloxacin	5	R
Tetracycline	30	R

^1^ Measured in activity units (U). S = susceptible and R = resistant.

**Table 2:** Viable counts for some of the individual treatments and combinations.

Heading Concentration (μg/mL)	Amikacin	*C. latifolia*	*C. sinensis*	Amk + *C. latifolia*	Amk + *C. Sinensis*
0.25	800,000	1.19	1.70	0.96	1.32
0.5	3132	1.52	1.21	0.83	0.94
1.0	0	1.40	1.70	0.28	0.33

Data are CFUs × 10^7^ except for amikacin (Amk).

**Table 3:** Antibacterial concentrations (μg/mL) used for evaluation of *E. coli* strain.

Cloramphenicol	Cefotaxime	Amikacin	*C. latifolia*	*C. sinensis*
1	7.5 × 10^−3^	0.25	0.25	0.25
2	15 × 10^−3^	0.5	0.5	0.5
4	60 × 10^−3^	1	1	1
8	0.118 × 10^−3^	2	2	2
	0.235 × 10^−3^	4	4	4
	0.47 × 10^−3^		96	96
			8.720 × 10^3^	8.420 × 10^7^
			8.720 × 10^7^	

**Table 4:** Antibacterial + EOs concentrations (μg/mL) used for combination essays.

Amikacin	*C. latifolia*	*C. sinensis*
1	1	-
0.5	1	-
0.25	1	-
1	-	1
0.5	-	1
0.25	-	1

## References

[1] Ventola C.L. (2015). The antibiotic resistance crisis: Part 1: Causes and threats. Pharm. Ther..

[2] Swamy M.K., Akhtar M.S., Sinniah U.R. (2016). Antimicrobial properties of plant essential oils against human pathogens and their mode of action: An updated review. Evid.-Based Complement. Altern. Med..

[3] Yap P.S.X., Yiap B.C., Ping H.C., Lim S.H.E. (2014). Essential oils, a new horizon in combating bacterial antibiotic resistance. Open Microbiol. J..

[4] Bakkali F., Averbeck S., Averbeck D., Idaomar M. (2008). Biological effects of essential oils–A review. Food Chem. Toxicol..

[5] Bassolé I.H.N., Juliani H.R. (2012). Essential oils in combination and their antimicrobial properties. Molecules.

[6] Arce A., Soto A. (2008). Citrus essential oils: Extraction and deterpenation. Tree For. Sci. Biotechnol..

[7] Nazzaro F., Fratianni F., De Martino L., Coppola R., De Feo V. (2013). Effect of essential oils on pathogenic bacteria. Pharmaceuticals.

[8] Costa R., Bisignano C., Filocamo A., Grasso E., Occhiuto F., Spadaro F. (2014). Antimicrobial activity and chemical composition of Citrus aurantifolia (Christm.) Swingle essential oil from Italian organic crops. J. Essent. Oil Res..

[9] Lopez-Romero J.C., González-Ríos H., Borges A., Simões M. (2015). Antibacterial effects and mode of action of selected essential oils components against Escherichia coli and Staphylococcus aureus. Evid.-Based Complement. Altern. Med..

[10] Kummer R., Fachini-Queiroz F.C., Estevão-Silva C.F., Grespan R., Silva E.L., Bersani-Amado C.A., Cuman R.K.N. (2013). Evaluation of anti-inflammatory activity of Citrus latifolia Tanaka essential oil and limonene in experimental mouse models. Evid.-Based Complement. Altern. Med..

[11] Lewis K. (2013). Platforms for antibiotic discovery. Nat. Rev. Drug Discov..

[12] Narang N., Jiraungkoorskul W. (2016). Anticancer activity of key lime, Citrus aurantifolia. Pharmacogn. Rev..

[13] Chaudhari S.Y., Ruknuddin G., Prajapati P. (2016). Ethno medicinal values of Citrus genus: A review. Med. J. Dr. DY Patil Univ..

[14] Jafarzadeh M., Arman S., Pour F.F. (2013). Effect of aromatherapy with orange essential oil on salivary cortisol and pulse rate in children during dental treatment: A randomized controlled clinical trial. Adv. Biomed. Res..

[15] Frassinetti S., Caltavuturo L., Cini M., Della Croce C., Maserti B. (2011). Antibacterial and antioxidant activity of essential oils from *Citrus* spp.. J. Essent. Oil Res..

[16] Singh P., Shukla R., Prakash B., Kumar A., Singh S., Mishra P.K., Dubey N.K. (2010). Chemical profile, antifungal, antiaflatoxigenic and antioxidant activity of Citrus maxima Burm. and *Citrus sinensis* (L.) Osbeck essential oils and their cyclic monoterpene, DL-limonene. Food Chem. Toxicol..

[17] Ruiz-Pérez N.J., González-Ávila M., Sánchez-Navarrete J., Toscano-Garibay J.D., Moreno-Eutimio M.A., Sandoval-Hernández T., Arriaga-Alba M. (2016). Antimycotic activity and genotoxic evaluation of Citrus sinensis and Citrus latifolia essential oils. Sci. Rep..

[18] Toscano-Garibay J., Arriaga-Alba M., Sánchez-Navarrete J., Mendoza-García M., Flores-Estrada J., Moreno-Eutimio M., Espinosa-Aguirre J., González-Ávila M., Ruiz-Pérez N. (2017). Antimutagenic and antioxidant activity of the essential oils of Citrus sinensis and Citrus latifolia. Sci. Rep..

[19] Zhu C., Liu Y., Hu L., Yang M., He Z.-G. (2018). Molecular mechanism of the synergistic activity of ethambutol and isoniazid against Mycobacterium tuberculosis. J. Biol. Chem..

[20] Kumar S.S., Ghosh A.R. (2019). Assessment of bacterial viability: A comprehensive review on recent advances and challenges. Microbiology.

[21] Thermofisher. LIVE/DEAD^®^BacLight™ Bacterial Viability and Counting Kit Manual. https://assets.thermofisher.com/TFS-Assets/LSG/manuals/mp34856.pdf.

[22] Thornton R.G.W., Gilmour L., Alsharif R. Evaluation of Yeast Viability and Concentration during Wine Fermenta-Tion Using Flow Cytometry. http://www.bdbiosciences.com/immunocytometry_systems/application_notes/.

[23] Moussaoui F., Alaoui T. (2016). Evaluation of antibacterial activity and synergistic effect between antibiotic and the essential oils of some medicinal plants. Asian Pac. J. Trop. Biomed..

[24] Robertson J., McGoverin C., Vanholsbeeck F., Swift S. (2019). Optimisation of the protocol for the LIVE/DEAD® BacLightTM bacterial viability kit for rapid determination of bacterial load. Front. Microbiol..

[25] Berney M., Hammes F., Bosshard F., Weilenmann H.-U., Egli T. (2007). Assessment and interpretation of bacterial viability by using the LIVE/DEAD BacLight Kit in combination with flow cytometry. Appl. Environ. Microbiol..

[26] Deng Y., Wang L., Chen Y., Long Y. (2020). Optimization of staining with SYTO 9/propidium iodide: Interplay, kinetics and impact on Brevibacillus brevis. BioTechniques.

[27] Rosenberg M., Azevedo N.F., Ivask A. (2019). Propidium iodide staining underestimates viability of adherent bacterial cells. Sci. Rep..

[28] Kjeldsen T.S., Sommer M.O., Olsen J.E. (2015). Extended spectrum β-lactamase-producing Escherichia coli forms filaments as an initial response to cefotaxime treatment. BMC Microbiol..

[29] Dhiman A., Nanda A., Ahmad S., Narasimhan B. (2012). In vitro antimicrobial status of methanolic extract of Citrus sinensis Linn. fruit peel. Chron. Young Sci..

[30] Das A., Vasundraa M., Vinotha V., Sanofer P., Bindhu J. (2019). Evaluation of Phytochemical Analysis and Antimicrobial Activity of Citrus Latifolia Peel Extract. Indian J. Public Health Res. Dev..

[31] Everton G.O., Teles A.M., Mouchrek A.N., Mouchrek Filho V.E. (2018). Extraction, chemical characterization and antimicrobial potency of essential oil of Tahiti lemon (Citrus latifolia Tanaka). Periódico Tchê Química.

[32] Adham A.N. (2015). Comparative antimicrobrial activity of peel and juice extract of citrus fruits growing in Kurdistan/Iraq. Am. J. Microbiol. Res..

[33] Tran T., Ngo T., Tran T., Bach L., Tran T., Huynh X. (2021). Comparison of volatile compounds and antibacterial activity of Citrus aurantifolia, Citrus latifolia, and Citrus hystrix shell essential oils by pilot extraction. IOP Conf. Ser. Mater. Sci. Eng..

[34] Guimarães A.C., Meireles L.M., Lemos M.F., Guimarães M.C.C., Endringer D.C., Fronza M., Scherer R. (2019). Antibacterial activity of terpenes and terpenoids present in essential oils. Molecules.

[35] Guo Q., Liu K., Deng W., Zhong B., Yang W., Chun J. (2018). Chemical composition and antimicrobial activity of Gannan navel orange (Citrus sinensis Osbeck cv. Newhall) peel essential oils. Food Sci. Nutr..

[36] Wishart D.S., Knox C., Guo A.C., Cheng D., Shrivastava S., Tzur D., Gautam B., Hassanali M. (2008). DrugBank: A knowledgebase for drugs, drug actions and drug targets. Nucleic Acids Res..

[37] CLSI (2017). Performances for Antimicrobial Susceptibility Testing.

